# Errors in Figure 3 and Omission in Additional Contributions

**DOI:** 10.1001/jamanetworkopen.2020.8117

**Published:** 2020-04-30

In the Original Investigation titled “Economic Evaluation of Chimeric Antigen Receptor T-Cell Therapy by Site of Care Among Patients With Relapsed or Refractory Large B-Cell Lymphoma,”^1^ published on April 6, 2020, there were errors in Figure 3 and an omission in Additional Contributions in the end matter.

In panel C of Figure 3, the label should have been “Adverse event management sensitivity analysis, base case (97.5% of patients have an AE).” In panel D of Figure 3, the label should have been “Adverse event management sensitivity analysis, scenario (50% of patients have an AE).” In panel D of Figure 3, the top line given as orange with the label “NASON” should have been blue with the label “Inpatient hospital.” The bottom line given as blue with the label “Inpatient hospital” should have been orange with the label “NASON.”

In the Additional Contributions section of the end matter, the listing omitted Adrienne Schreiber, BA, of The Lockwood Group (Stamford, Connecticut). This article has been corrected.^1^

## References

[1] Lyman GH, Nguyen A, Snyder S, Gitlin M, Chung KC Economic evaluation of chimeric antigen receptor T-cell therapy by site of care among patients with relapsed or refractory large B-cell lymphoma. JAMA Netw Open. 2020;3(4):e202072. doi:10.1001/jamanetworkopen.2020.2072 32250433 PMC7136832

